# A genome-wide association study identifies only two ancestry specific variants associated with spontaneous preterm birth

**DOI:** 10.1038/s41598-017-18246-5

**Published:** 2018-01-09

**Authors:** Nadav Rappoport, Jonathan Toung, Dexter Hadley, Ronald J. Wong, Kazumichi Fujioka, Jason Reuter, Charles W. Abbott, Sam Oh, Donglei Hu, Celeste Eng, Scott Huntsman, Dale L. Bodian, John E. Niederhuber, Xiumei Hong, Ge Zhang, Weronika Sikora-Wohfeld, Christopher R. Gignoux, Hui Wang, John Oehlert, Laura L. Jelliffe-Pawlowski, Jeffrey B. Gould, Gary L. Darmstadt, Xiaobin Wang, Carlos D. Bustamante, Michael P. Snyder, Elad Ziv, Nikolaos A. Patsopoulos, Louis J. Muglia, Esteban Burchard, Gary M. Shaw, Hugh M. O’Brodovich, David K. Stevenson, Atul J. Butte, Marina Sirota

**Affiliations:** 1 Institute for Computational Health Sciences, University of California, San Francisco, 94143 CA USA; 2 Department of Pediatrics, University of California, San Francisco, San Francisco, CA USA; 3 Department of Pediatrics, Stanford University School of Medicine, Stanford, CA USA; 4 Department of Genetics, Stanford University School of Medicine, Stanford, CA USA; 5 Institute for Human Genetics, University of California, San Francisco, San Francisco, CA USA; 6 Inova Translational Medicine Institute, Inova Health System, Falls Church, VA USA; 7 Department of Population, Family and Reproductive Health, Center on the Early Life Origins of Disease, Johns Hopkins University Bloomberg School of Public Health, Baltimore, MD USA; 8 Cincinnati Children’s Hospital Medical Center, Cincinnati, OH USA; 9 Department of Biostatistics, University of California, San Francisco, CA USA; 10 Systems Biology and Computer Science Program, Ann Romney Center of Neurological Diseases, Department of Neurology, Division of Genetics, Brigham & Women’s Hospital, Boston, MA USA; 11 Harvard Medical School, Boston, MA USA; 12 Program in Medical and Population Genetics, Broad Institute of MIT and Harvard, Cambridge, MA USA

## Abstract

Preterm birth (PTB), or the delivery prior to 37 weeks of gestation, is a significant cause of infant morbidity and mortality. Although twin studies estimate that maternal genetic contributions account for approximately 30% of the incidence of PTB, and other studies reported fetal gene polymorphism association, to date no consistent associations have been identified. In this study, we performed the largest reported genome-wide association study analysis on 1,349 cases of PTB and 12,595 ancestry-matched controls from the focusing on genomic fetal signals. We tested over 2 million single nucleotide polymorphisms (SNPs) for associations with PTB across five subpopulations: African (AFR), the Americas (AMR), European, South Asian, and East Asian. We identified only two intergenic loci associated with PTB at a genome-wide level of significance: rs17591250 (P = 4.55E-09) on chromosome 1 in the AFR population and rs1979081 (P = 3.72E-08) on chromosome 8 in the AMR group. We have queried several existing replication cohorts and found no support of these associations. We conclude that the fetal genetic contribution to PTB is unlikely due to single common genetic variant, but could be explained by interactions of multiple common variants, or of rare variants affected by environmental influences, all not detectable using a GWAS alone.

## Introduction

Preterm birth (PTB), the delivery of an infant prior to 37 weeks of gestation, is a significant determinant of infant morbidity and mortality. Globally, in 2010 an estimated 11% of births, totaling 15 million were delivered preterm^1,2^. In the US, PTB occurs in nearly 10% of all births. Infants born preterm are at risk for a variety of long-term adverse outcomes, such as respiratory illness, blindness, and cerebral palsy, with associated complications resulting in nearly one million deaths each year. PTB is the top cause of death in children under-five years of age^3^. Although survival for most children born premature has improved considerably, they remain at increased risk for a variety of severe neurodevelopmental, gastrointestinal, and respiratory complications, many of which extend well beyond the neonatal period and contribute to lifelong challenges for individuals and their families as well as to burdensome economic costs to society.

While approximately 20% to 30% of PTBs are medically indicated or performed to minimize potential complications from delivery, the vast majority of PTBs are spontaneous, caused by either preterm labor or preterm premature rupture of membranes (PPROM)^4^. The exact mechanism of spontaneous PTB is unknown, though a multitude of social^5,6^, environmental^7,8^, and maternal factors, such as a shortened interpregnancy interval^9–11^, young maternal age^1,2,12–15^, extremes of body mass index^16–18^, and increased stress levels, have been implicated. Various observations, such as the tendency for recurrent PTBs^19–22^ and the increased risk of preterm delivery for women who themselves were born preterm^23,24^, suggest a heritable component to the risk of PTB^25^. Twin studies estimate that maternal genetic variants account for approximately 27% to 36% of the incidence of PTB^26,27^. A recent study estimated that 11% of variation is due to fetal genetic effects^28^. A follow up study which focused on quantifying the extent that heritable factors and environmental exposures predict the timing of birth and explain differences between racial groups, found a significant effect in individuals with European ancestry, but not African Americans^29^. Furthermore, PTB rates vary among races and ethnicities, with elevated frequencies observed in African Americans when compared to European Americans^19,22,30,31^, and environmental and socioeconomic factors alone do not readily account for these differences^32^. Currently, it is unclear how the genetics of PTB differ across racial and ethnic groups.

Despite the evidence for contribution of genetics to the risk of PTB, to date very few reproducible associations have been identified through the genome-wide screens conducted for PTB. The majority of approaches adopted thus far have focused on candidate genes in pathways previously associated with PTB, such as immunity and inflammation^33–37^. The study of the genetics of PTB is further complicated by the likely complex interactions of genes and the environment within these highly interactive causal pathways. Linkage and rare-variant analyses as well as a few genome-wide association studies (GWAS) have been performed to study the genetic factors contributing to the risk of PTB^38^, however, replication efforts have been limited. The challenge in carrying out such analyses is further complicated by possible effects arising from maternal and fetal genetic influences.

Several genome-wide linkage-based strategies have been used to identify maternal and fetal genetic variants on chromosomes 15 and X that contribute to PTB in Finnish multiplex and nuclear families^39,40^. Bream *et al*. used candidate gene linkage analyses to identify a set of mutations potentially linked to fetal-mediated PTB and another set of variants linked to maternal-mediated PTB based on 257 families with a family history of PTB^41^. A study by Chittoor *et al*. examined the susceptibility of Mexican Americans to PTB using a linkage analysis across 1,439 individuals and found a significant linkage signal for a region on chromosome 18 containing 52 potential candidate genes^42^. Whole exome sequencing of a limited set of maternal genomes from European families revealed an over-representation of rare variants in the complement/coagulation factor cascade^43^. There have been several large GWAS that have identified variants associated with low birth weight^44–46^, but only a limited number have focused on spontaneous PTB. Evolutionary approaches have been applied to identify genes involved in human birth timing^47^, and a recent study of mother-infant pairs found two single nucleotide polymorphisms (SNPs) associated with early spontaneous PTB using data collected by the Genomic and Proteomic Network (GPN) for Preterm Birth Research. Neither of these SNP associations, however, could be replicated in independent cohorts^48^. A very recent large scale study identified several genomic regions in European mothers who have delivered preterm^49^. The aforementioned work as well as other studies to date have focused on maternal effects, and not on fetal genetic influences. In the current study, we focused on fetal associations in several populations. Finally, there are several databases summarizing up-to-date findings of the genetics of PTB; however, high-value genetic candidates for diagnostic and therapeutic targets for further study have not been identified^50,51^.

In addition to the evidence for the role of genetic factors in PTB, there are pronounced and persistent racial and ethnic disparities observed in the rates of PTB, with significantly higher rates observed in African Americans^52^. The vast majority of genetic studies to date have been performed in European populations, resulting in poor generalizability of these findings to minority populations^53^. Although the factors that underlie this disparity remain elusive, they likely involve complex interactions between genetics, neighborhood-level environmental exposures, and infection and inflammation^54^. A recent study by Hong *et al*. showed that maternal COL24A1 variants have a significant genome-wide interaction with maternal pre-pregnancy overweight/obesity on PTB risk in a population of African Americans^55^. Nonetheless, even after decades of basic science research and public health initiatives, this racial disparity remains poorly understood and relatively unchanged, and specific fetal genetic markers are yet to be found.

In this study, we performed a large ancestry-informed GWAS of over 1,300 infants that were born preterm. To our knowledge, this is one of the largest reported cross-ethnic GWAS performed for this phenotype. Leveraging publicly available data, we were able to employ a large cohort of over 12,000 individuals to serve as a control for our analyses. Using this approach, after extensive filtering and quality control, we identified only two intergenic variants that are statistically significantly associated with PTB and show some evidence in several independent cohorts.

## Results

**Figure 1:**
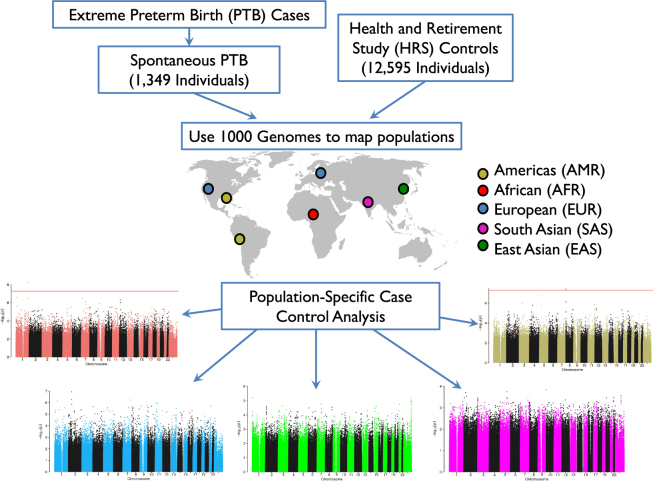
Analysis Workflow. We started with a set of PTB cases and chose to focus on those that were born preterm spontaneously (1,349 individuals). We also assembled a large group of control individuals who have a very low chance of being born preterm (12,595 individuals). We used the 1,000 Genomes dataset in order to map both cases and controls to five populations: Americas (AMR), African (AFR), European (EUR), South Asian (SAS), and East Asian (EAS). Finally, we carried out case control analysis in each of the five populations. World map image adapted from Wikipedia: https://upload.wikimedia.org/wikipedia/commons/1/17/BlankMap-World-noborders.png.

We carried out a genome-wide association analysis comparing 1,349 extreme preterm infants delivered between 25 and 30 weeks of gestation and documented clinically as spontaneous PTB in the years 2005 to 2008 (Figure S1), with 12,595 ancestry-matched controls. The workflow for this analysis is shown in Fig. 1.

The 1,349 preterm cases were defined as spontaneous PTB deliveries based on birth certificate records. These cases were previously studied in an effort to identify genetic markers for PTB-associated chronic lung disease^56^. The ancestry-matched control population was obtained from the Health and Retirement Study (HRS)^57^, the vast majority of whom are older than 55 years of age at the time of recruitment, which started in 1992 (Table S1 and Figure S3). In the early 1990s, major advances in neonatal care, led by the introduction of surfactant therapy^58^, greatly increased the likelihood of survival for infants born before 30 weeks^59–62^. Since all of the controls were retirees born well before 1990, we assumed that they were very unlikely to have been born extremely preterm (before 30 weeks), and, therefore, can serve as an appropriate control population for our genome analyses.

**Figure 2:**
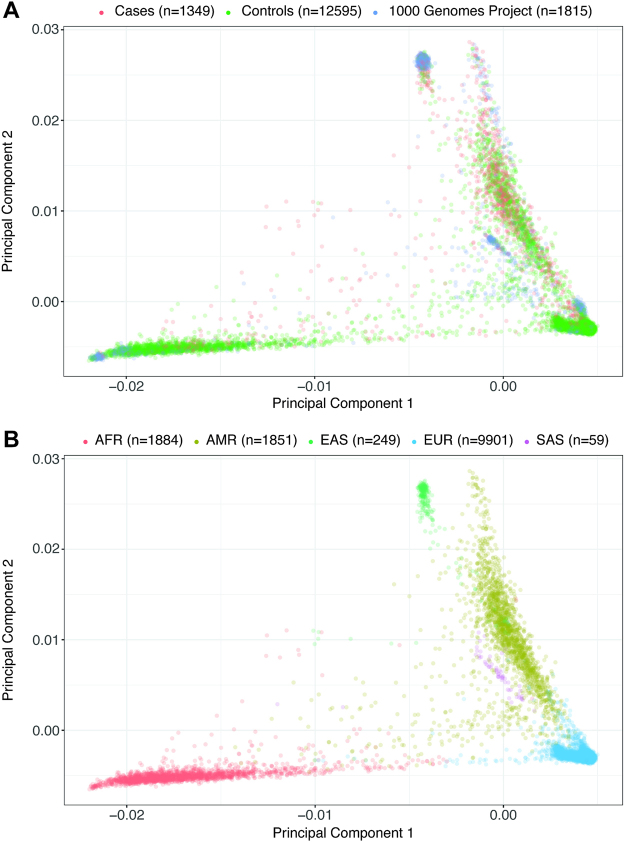
Principal components of genetic ancestry across 15,734 individuals. (**A**) This is a PCA plot showing the first and second principal components of genetic ancestry colored by dataset for the three datasets in our analyses. The PTB cases are shown in red, HRS controls shown in green, and 1,000 Genomes Project shown in blue. (**B**) This is a PCA plot similar to the one above, however colored by the five populations that our cases and controls were stratified into.

To account for differences in population substructures between the cases and controls, we used 1,815 individuals from Phase 3 of the 1,000 Genomes Project as an anchor point to match cases and controls (Fig. 2, Figure S4). Cases and controls were classified into one of the five population cohorts based on their relative ancestral distance to individuals in the 1,000 Genomes Project^63^ (See Methods): African (AFR), the Americas (AMR), East Asian (EAS), European (EUR), and South Asian (SAS) using principle component analysis (Table 1).

**Table 1:** Number of spontaneous PTB cases and HRS controls in the presented GWAS.

Population	Sex	PTB Cases	HRS Controls
EUR (n = 9,890)	Female	121	5,575
	Male	139	4,055
	Total	260	9,630
AFR (n = 1,874)	Female	103	1,078
	Male	87	606
	Total	190	1,684
AMR (n = 1,847)	Female	337	672
	Male	408	430
	Total	745	1,102
EAS (n = 249)	Female	72	77
	Male	59	41
	Total	131	118
SAS (n = 59)	Female	7	17
	Male	16	19
	Total	23	36

We tested 2,015,750 SNPs for their association to the spontaneous PTB phenotype in each of the five population cohorts separately, with sex and the first ten principal components of genetic ancestry as covariates in the model. The genomic inflation factor (*λ*)^64^ was 1.02, 1.05, 1.04, 1.02, and 1.13 for AFR, AMR, EAS, EUR, and SAS subpopulations, respectively, suggesting little evidence for residual population stratification. The Q-Q plots for these two populations show a well-calibrated distribution of p-values with the majority of values falling on the diagonal (Figure S5).

**Figure 3:**
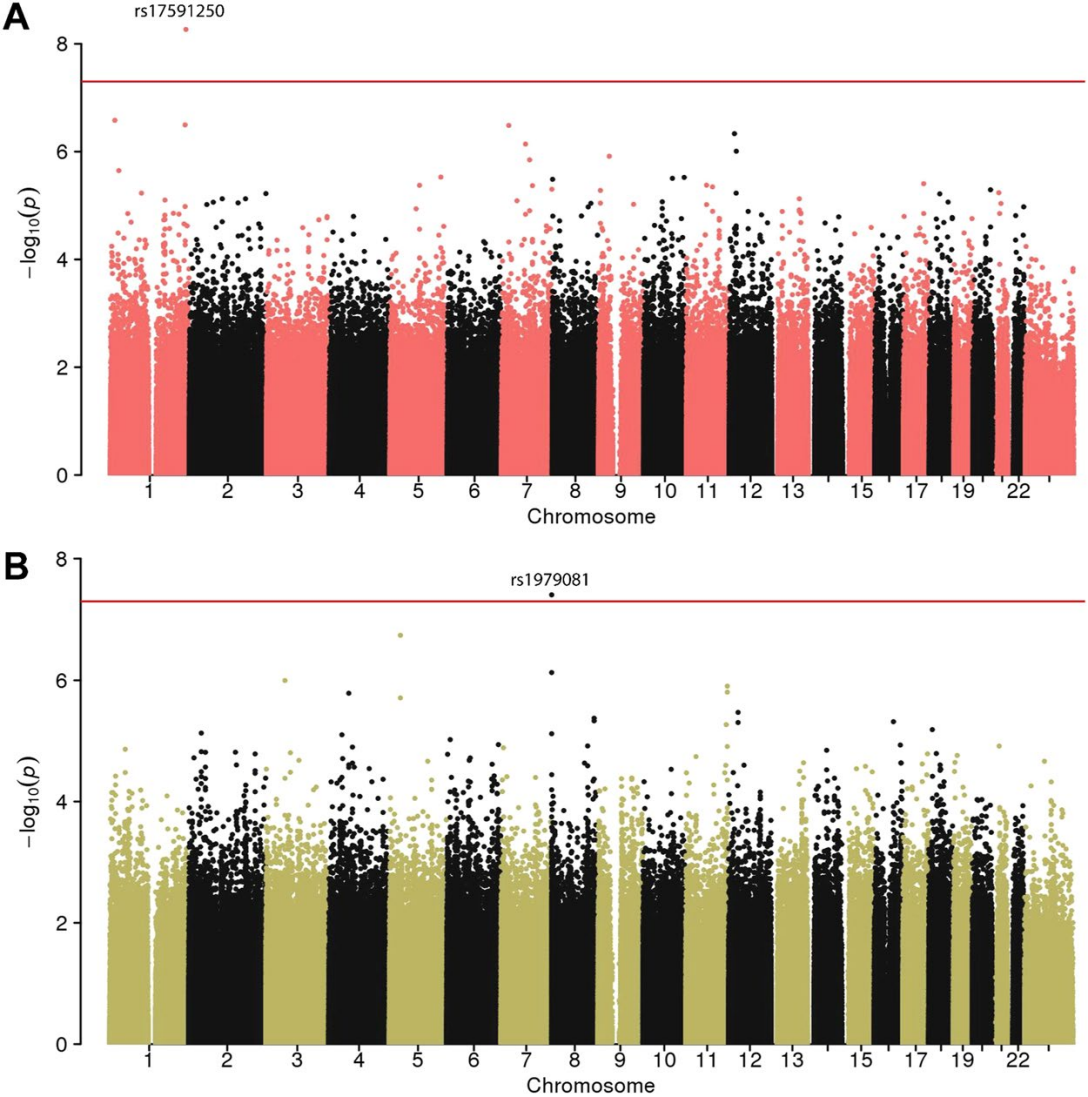
Manhattan Plots in the African (AFR) and Americas (AMR) populations. R (**A**) and AMR (**B**) populations. The chromosomes are on the x-axis and the -log (P-value) is shown on the y-axis. Genome-wide significant variants are shown above the dotted line.

Overall, we identified two loci associated with spontaneous PTB at a genome-wide level of significance (Table 2), both are intergenic loci - one variant in the AFR population and one in the AMR population (Fig. 3). The analyses in the other three (i.e., EAS, EUR, and SAS) populations did not result in any significant genome-wide associations (Figure S6).

**Table 2:** SNPs with genome-wide significant associations with spontaneous PTB. OR and p-values for the other discovery and validation cohorts are given in Table S2.

CHR	SNP	BP	Minor allele	OR	P-value	POP	Genes (distance in bp)	Case genotype counts	Control genotype counts	Validated
1	rs17591250	238386465	G	2.814	4.55E-09	AFR	RP11-136B18.1 (45329)	GG:2,GA:52	GG:3,GA:204	G,T
							RP11-136B18.1 (45329)	AA:127	AA:1451	
8	rs1979081	334288	A	0.566	3.72E-08	AMR	FAM87 (864)	AA:19,AG:147	AA:38,AG:352	M,I
							FBXO25 (22520)	GG:535,00:35	GG:665,00:29	

OR - odds-ratio. POP - The population in which the association was identified. Missing genotypes are labeled as ‘00’. M, I, G, T P-value <0.05 in external cohort FIN-mothers, FIN-infants, GALA II, ITMI respectively.

The most significant variant in the AFR population corresponded to rs17591250 (OR = 2.814, P = 4.55E-09), an intergenic SNP on chromosome 1. This variant is located between the pseudogene YWHAQP9 (distance ~100 Kb) and RP11-136B18.1 gene (distance ~ 45 Kb). The minor allele frequency of this variant in our AFR case-control cohort of 0.072 is similar to the one on gnomAD^65^ of 0.07804 for the AFR population and slightly higher than the one reported in dbSNP (0.041)^66^. The second significant variant was found in the AMR population and corresponds to rs1979081 (OR = 0.5656, P = 3.72E-08) (Table 2). This variant is also intergenic, located on chromosome 8, 864 bp upstream to FAM87A (Family With Sequence Similarity 87 Member A) gene and ~22 Kb from FBXO25 (F-Box Protein 25) gene.

To further test whether these results are robust, we imputed the region of each variant after removing the significant variants, and tested whether the association was recovered (see Methods). Both variants were imputed with high certainty (>0.99). In the AFR population, rs146565706, a variant 1931bp from rs17591250, also had a significant association to PTB (P = 3.91E-09). We also identified rs17591327 (560 bp away) with the same P-value as rs17591250 (Figure S7). In the AMR imputation analysis, the variant rs1979081 was not fully recovered, but the region around the variant has several other loci with elevated significance (Figure S7) pointing to the potential association of the region with PTB.

We have queried several replication datasets to see whether our findings are replicated in independent cohorts. We recognize that none of the existing replication datasets that we used were well matched to our discovery cohort based on phenotypic and demographic parameters. The replication results are summarized in Table S2. We analyzed 307 PTB cases and 2,826 controls from the Latino population from the Genes-environments and Admixture in Latino Americans babies (GALA II) study^67^ and found that variant rs17591250 on chromosome 1 was modestly significant (OR = 0.7, P = 0.02775;, however, we observed a flip in the odds ratio (OR) for this population. This could be due to the difference in minor allele frequencies for this variant in different ethnic groups (AFR = 0.04 vs. EUR = 0.20 and AMR = 0.19 dbSNP build ID: 123/150^66^). We did not observe significant association for rs1979081 in this cohort.

Another validation cohort was obtained from Inova Translational Medicine Institute (ITMI, Inova Health System, Falls Church, VA). This cohort consists of 800 trios (mother, father, and infant) on whom whole genome sequencing was performed. In this cohort, cases were defined as early preterm (≤34 weeks of gestation) and controls as full-term (≥37 weeks of gestation). The analysis of the infants’ genomes was carried out separately in the AFR and the EUR subpopulations (see Methods). We found rs17591250 was nominally significant in the EUR population (OR = 0.563, P = 0.04842), however we again observed a flip in the OR, which could be explained by the difference in allele frequencies in the different populations. In the ITMI cohort, we also examined variants within 1Kb of the associations and were able to identify regional support for the association (Table S3). We did not observe significant association for rs1979081 in this cohort, but we saw some regional support. The fact that the p-values are close to 0.05 and effect directions are different in the validation sample indicate a high uncertainty of the estimation.

In addition, we examined maternal genomic influences in these regions using several additional existing cohorts. The FIN cohort is composed of over 800 European mother/child pairs. We queried this cohort for variants within 1Kb of the associations (see Methods). In the mothers’ cohort, 6 variants within the 1Kb window around rs1979081 had an association P-value <0.05, and OR ranged between 1.7515 and 1.8143 and in the babies’ cohort a single variant was statistically associated (OR = 1.3966, P = 0.04373) (Table S4). While we observe some statistical association in this region, the evidence is weak, which might point to the fetal vs. maternal genetic effects on PTB. We did not observe any significant association for the region surrounding rs17591250 in this cohort.

Finally, we carried out an association analysis using the Boston Birth Cohort (BBC) as an independent cohort. This cohort consists of 698 African-American mothers who had preterm deliveries and 1,035 mothers of term controls (See Methods). We were not able to validate our findings in this cohort, which might point to the fetal vs. maternal effects on these genetic associations.

## Discussion

In this report, we present the largest reported genome-wide study of early spontaneous PTB with over 1,300 cases of early spontaneous PTB compared with 12,000 ancestry-matched control individuals obtained from a separate independent study. We identified only two loci that were significantly associated with PTB in the AFR and AMR cohorts.

The most significant AFR variant discovered was rs17591250, an intergenic variant on chromosome 1 between the pseudogene YWHAQP9 and RP11-136B18.1 gene. It is ~ 300 Kb away from the Zona Pellucida Glycoprotein 4 (ZP4) gene. ZP4 is an extracellular matrix protein that surrounds the oocyte and early embryo. The most significant AMR variant found was rs1979081. The variant is also intergenic on chromosome 8 between FAM87A and FBXO25. Two other genes are ~130 Kb farther: ZNF596 (Zinc Finger Protein 596) and TDRP (Testis Development Related Protein). TDRP is known to be highly expressed in the placenta^68,69^. FBXO25 is mostly expressed in the testis. FAM87A and ZNF596 were found to be highly expressed in the brain and testis and FAM87A was also highly expressed in the pancreas^68,69^. This SNP is a known eQTL in different tissues for the lncRNA RP11-91J19.4 gene (Consortium ^68^), which is highly expressed in the brain. The two significant variants found are in intergenic regions, and we cannot yet ascribe any direct effect from these SNPs.

There are several limitations of this study that need to be noted. While the case and control populations were genotyped on the same platforms and the cases and controls were matched based on their ancestry using a clustering approach, the study design was not optimal. We have carried out extensive quality control to eliminate any potential batch effects, but ideally the cases and controls should emerge from the same population at risk and be genotyped in a single pass to avoid potential bias and batch effects. Also, once the cases were stratified by the five ancestral populations, the number of cases in each ancestry population was relatively small. Due to the small sample size for the SAS and EAS cohorts in particular, and the extreme unbalanced case-control populations for the EUR, we were not able to identify any variants that passed the genome-wide significance threshold for an association with PTB. We used strict filtering tests and thresholds to remove variants with low-call scores, missing or differentially missing in cases and controls; and therefore, we might not have been able to identify some relevant associations. We assumed that the HRS was a reliable cohort for use as a control set for the PTB phenotype due to the low survival of preterm babies prior to 1990. Other phenotypic factors such as age^70,71^, BMI^16^, and others need to be considered in future analyses. Finally, we define PTB here as a binary trait, while another approach would be to model gestational age at delivery as a continuous phenotype.

The limitations of our validation cohorts should also be recognized. None of the cohorts ideally mimic our discovery population, which is focused on early spontaneous PTBs. The validation cohorts are not as strictly phenotyped, are sampled from diverse ethnic backgrounds and several of them were not collected to study preterm birth specifically. The validation cohorts are also limited by sample size. Finally, while several of the cohorts contain a limited number of fetal samples, some of them focus on maternal effects, and albeit sharing half of the DNA with the fetus, these can only be used as supporting evidence and cannot be interpreted as validation. Much more rigorous validation of our findings with better matched cohorts is needed.

Using genotype approaches (i.e., GWAS), we were only able to identify markers (e.g., tagSNPs) associated with PTB and not necessarily any causal variants. Fine mapping gene sequencing or use of a whole genome sequencing approaches are needed to exactly locate causal mutations that might be responsible for the phenotype. With numerous sequencing and genotyping efforts underway, we hope to leverage some of these resources for further validation and analyses. Finally, we have focused our work here on identifying potential genetic risk variants associated with PTB; however, we recognize that this is only a part of the complete picture. In the current analysis, environmental factors were not captured even though previous work had shown that these factors might have an effect^36^. Future studies should be designed to allow us to explore pertinent environmental exposures and examine gene-environment interactions in the context of the phenotype of interest.

While the sample size of this study is considerable, it does seem like the study is still underpowered to identify robust fetal genome-wide associations and additional studies are needed. Our results highlight only two significant variants, though of relatively small effect, throughout the genome suggesting that there are more elusive sources of heritability to consider for PTB. First, the GWAS we performed is best powered to find common variants of small effect in large populations^72^. However, human genomes are rich in structural diversity outside of SNPs that are individually rare, but collectively common in the human population^73,74^ and result in large genomic spans of deletions, duplications, and inversions that impact genes with large effects. Second, differences in genetic architecture reflect the complex, often opposing effects of selection, population history, migration and mutation rates^75^, and the cumulative genetic effects of these influences are also likely to be common in complex phenotypes^76,77^. Third, and for PTB in particular, currently unknown gene-environment (GxE) interactions most likely contribute to the phenotype. These and other shortcomings^75^ can be addressed with more sophisticated models of disease genetics of PTB that depend on deeper phenotyping and sequencing of subjects to identify the interaction of environmental triggers and rare variants that likely contribute to the phenotype.

In conclusion, we have presented an ancestry-informed GWAS of PTB including over 1,300 early spontaneous PTB cases and over 12,000 ancestry-matched controls across five populations. We leveraged publicly-available genotypic data to identify a large control group that is highly likely to be depleted for the phenotype of PTB, and we also mapped cases and controls based on their ancestry lineage to carry out population-specific case-control analyses. The analytical approach we present here can be extended to study other phenotypes of interest. Finally, we identified two different variants associated with PTB in two different populations. Neither of the associations we identified has previously been linked to PTB to our knowledge, and the biological significance of these associations has yet to be determined. While additional validation is needed, we anticipate that there might be other variants in these genomic regions that may contribute to a wide range of varying capacities for expression among the general population that singly or in combination might contribute to individual differences in response to environmental stressors and the risk of PTB.

## Methods

### Case and control cohorts

The 1,349 cases used in this study were obtained from the California Perinatal Quality Care Collaborative (CPQCC) database during the 2005 to 2008 calendar years. Inclusion criteria included gestational age 25 to 29 and 6/7 weeks (Figure S1), birthweight less than 1500 g (Figure S2), and spontaneous delivery defined by PPROM, premature labor, or tocolysis as recorded on birth certificates. These cases were previously analyzed as part of a GWAS on bronchopulmonary dysplasia and additional inclusion criteria are detailed in this prior study^56^.

The 12,595 controls used in this analysis were obtained from the University of Michigan HRS (http://hrsonline.isr.umich.edu/gwas), a longitudinal survey of Americans who ranged from 50 to 80 + years old at the time of data collection (Table S1; Figure S3). Data were downloaded from dbGap (accession pht002614.v1.p1) on January 2016.

### Genotyping

For the cases, genomic DNA was extracted from bloodspots^78^ and genotyped using the HumanOmni2.5-4 v1 BeadChip (Illumina, San Diego, CA), as previously described^56^. For the controls, saliva was collected and genotyped by the NIH Center for Inherited Disease Research using the same Human Omni2.5-4 v1 BeadChip methodology, as previously described.

We merged raw intensities data (*.idat) files of cases and controls and carried out joint calling, to reduce batch effects. Clustering and genotyping were performed using GenomeStudio software 2011.1 (Illumina, 2011). Quality control procedures were performed, including filtering by call rate, mismatch between observed and reported gender and possible gender abnormalities as described in Wang *et al*.^56^, and tri-allelic and ambiguous (AT/CG) SNPs were removed. Overall, a total of 2,015,750 SNPs were used in our downstream analyses.

### Ancestry matching of cases and controls

To minimize potential confounding factors introduced by population stratification, we matched cases and controls to defined population cohorts as provided by the 1,000 Genomes Project. We performed PCA on our cases, controls, and 1,815 individuals from the 1,000 Genomes Project using a subset of 102,008 common (minor allele frequency greater than 5%) autosomal SNPs that are in Hardy-Weinberg equilibrium. These SNPs are also relatively independent, with pairwise linkage disequilibrium values (R2) less than 0.20. Next, utilizing population labels provided for individuals in the 1,000 Genomes Project and the top 10 principal components, we inferred population labels for our cases and controls using the k-nearest neighbor algorithm^79^ (Fig. 2 and Figure S4).

### Association analysis

We analyzed potential associations between 2,015,750 SNPs and spontaneous PTB status using logistic regression in PLINK v1.90b3.42^80^. We stratified our analyses by population (inferred by principal components of genetic ancestry) and used the top ten principal components of genetic ancestry and sex as covariates. Variants were filtered out in case of missing calls >5% either in cases or controls, or P-value of differential missingness between cases and controls <0.001, or Hardy-Weinberg equilibrium <0.001 in cases and controls separately or jointly, or MAF <0.001. Outlier samples were filtered out if standard deviation using first 10 first principal components was larger than 6. This was performed iteratively re-computing PCA and removing outliers until no more outliers were detected using EIGENSTRAT software^81^. Sample outliers with identity by descent >0.2 were removed as well. We confirmed the results visually for the lack of population structure by plotting cases, control and matching 1,000 Genome Project samples by the top two principle components (Figure S8)

### Manual inspection of variant intensities

In order to ensure the quality of the genotyping data, following joint calling of cases and controls using GenomeStudio software, we manually inspected the variant calling and clustering for the two variants that we identified as significantly associated in the study (Figure S9).

### Manhattan plots

Manhattan plots were generated in R version 3.3.1 using qqman library version 0.1.2^82^. Significance line was plotted at the level of genome-wide significant threshold of 5E-8. Zoomed-in manhattan plots were generated using the LocusZoom web tool^83^.

### Imputation

For each one of the two significant variants found, we extracted the genotyping calls from ± 1mbp excluding the variant of interest. Prephasing was done with EAGLE2 v2.3.1^84^ and imputation using IMPUTE2^85^ and 1000 G V3 as a reference.

### Replication cohorts

Validation of associations has been carried out in three independent cohorts: (i) The Study of African Americans, Asthma, Genes and Environments (SAGE II)^67^ (ii) Genes-environments and Admixture in Latino Americans babies (GALA II) study^67^ (iii) Inova Translational Medicine Institute’s (ITMI) PTB study cohort.

#### SAGE II cohort

The Study of African Americans, Asthma, Genes, and Environments (SAGE II) is a gene-environment interaction study of asthma in African-American children in the USA initiated in 2006. Over 1,500 Individuals aged 8–21 years were collected from the San Francisco Bay during 2006–2013. Available genotyping data cover 114 PTB cases (labor prior to week 36) and two sets of controls: 996 controls born non-preterm (after 36 weeks of gestation) and a subset of 225 individuals who were born full-term (≥40 weeks of gestation). DNA was extracted from whole blood samples and genotyped with the Affymetrix Axiom LAT1 array^86^.

#### GALA II cohort

The GALA II is a case-control study using protocols and questionnaires as described in Nishimura *et al*.^67^. Subjects were recruited from five urban study centers across the mainland US and Puerto Rico (n = 7,683). Genome-wide SNP genotypes are from the Axiom^TM^ LAT1 array (Affymetrix, >800,000 SNPs). Latino children were recruited from the San Francisco Bay Area, Chicago, Houston, New York City, and Puerto Rico. Although the original dataset was collected to study asthma and drug response in these children, several questions related to birth timing were part of the original questionnaire, including whether the child was born early, on time or late and how many weeks early or late. Using the questionnaire data, we were able to identify PTB case and control populations in order to investigate the genetic determinants that are responsible for the observed phenotype of PTB. The 307 cases here are individuals who reported being born between 25 and 36 weeks of gestation. We used two set controls: the first one includes 2,826 individuals who were born non-preterm and the second is a subset of 309 individuals who were born full-term (≥40 weeks of gestation).

#### ITMI cohort

A validation cohort was obtained from Inova Translational Medicine Institute (ITMI), Inova Health System, Falls Church, Virginia. This cohort consisting of 800 trios (mother, father, and infant) on which whole genome sequencing was performed. In this cohort, cases were defined as early preterm (≤34 weeks gestation) and controls as full-term (≥37 weeks gestation). The analysis of the infants’ genomes was carried out separately in the AFR (31 early preterm cases and 53 controls) and the EUR (82 early preterm cases and 249 controls) sub-populations.

**Study participants and whole genome sequencing**: Trios comprised of neonates and both parents were enrolled in Inova Translational Medicine Institute’s “Molecular Study of Pre-term Birth”^87^. Gestational ages of the infants ranged from 22 to 41 weeks. Whole genome sequencing of peripheral blood samples collected from each participant was performed at Complete Genomics (Mountain View, CA), and sequencing data were processed as described^87^. The genomic data were used to assign ancestry using principal components analysis with the 1,000 Genomes Project data as reference as described above.

**Variant filtering**: Filtering required genotypes to be fully called with GQ ≥42, allele balance >0.225, read depth ≥9, and call rate ≥80%, with variants annotated as VQLOW excluded. Vt^88^ was used for multi-allelic variant decomposition, block substitution decomposition, and variant normalization. The resulting genotype calls were converted to bed format with PLINK v1.90b2a.

**Association analyses**: For each ancestry group, association tests were performed on early preterm (≤34 weeks gestation) vs. full-term (≥37 weeks gestation) infants from singleton births. The association testing was performed with a logistic model using gestational age, gender, and the eigenvectors from the ancestry-based PCA as covariates as described above. The AFR group was comprised of 84 neonates, 31 preterm cases and 53 controls. The EUR group had 331 infants, 82 cases and 249 controls. The association analyses and genotype frequencies were computed with PLINK v1.90b2a. We have also examined variants within 1Kb of the associations.

### Additional maternal cohorts

In addition to replication cohorts, we looked at maternal genetic influences in the regions we found in two additional cohorts (i) The Boston Birth Cohort (BBC); (ii) The Finnish cohort (FIN).

#### BBC cohort

The GWAS of PTB in the Boston Birth Cohort (BBC) is funded by NICHD (R01 HD041702, Principal Investigator: Wang, Xiaobin). This study includes 698 mothers with PTB (ranging from 230/7 to 366/7 weeks of gestation) and 1035 mothers with term births (370/7 to 430/7 weeks). All of these mothers are African Americans. The preterm and term mothers were matched on maternal age (±5 years), parity and years of delivery (in frequency). Maternal DNA was extracted from white blood cells and genotyped using the Illumina HumanOmni2.5 array. SNP data filtering followed the NIH GENEVA consortium published protocol. The recruitment and characteristics of the BBC has been described previously^89,90^. Eligible mother-infant pairs were recruited 1 to 3 days post-delivery. Mothers who delivered singleton livebirths were included in the study. Pregnancies that were a result of *in vitro* fertilization or multiple gestations, fetal chromosomal abnormalities or major birth defects were excluded. Analyses used the imputed SNPs, with genotype dosage as the predictor. The analysis was done using SNPTEST2 (-Frequentist function), with adjustment of batch, the first three principle components from PCA, infant’s gender and maternal age and parity.

#### FIN cohort

The Finnish cohort (FIN) cohort includes genotypes of 817 babies (253 cases) and 888 mothers (334 cases) and gestational age on labor^49^. Mother/child pair samples were collected from the Helsinki (southern Finland) University Hospitals between 2004 and 2014. Regression to gestational week on labor was performed for babies and mothers separately using imputed allelic dosage data assuming additive allelic effects. Maternal age and infant gender were included as covariates. We queried this cohort for variants within a 1Kb of our associations.

### Data Sharing and Availability

The complete summary statistics and results files are available through ImmPort (http://www.immport.org/) SDY1205, 10.21430/M37N6PJEQT. Results of the analysis and interactive visualization is available as an R Shiny App at http://comphealth.ucsf.edu/ptb_gwas.

### Ethical statement

The controls from this study came from a publicly available dataset called the Health and Retirement Study^57^. The cases were obtained from California blood spots and are restricted to data sharing. California has determined that all requests for the use of California Biobank Program biospecimens for research studies will need to seek an exemption from NIH or other granting or funder requirements regarding the uploading of study results into an external bank or repository (including into the NIH dbGaP or other bank or repository). This applies to any uploading of genomic data and/or sharing of these biospecimens or individual data derived from these biospecimens. Such activities have been determined to violate the statutory scheme of the California Health and Safety Code Sections 124980(j), 124991(b), (g), (h) and 103850(a) and (d), which protect the confidential nature of biospecimens and individual data derived from biospecimens.

## Electronic supplementary material

Supplementary Information
