# Supplementary material for: A genome-wide association study identifies only two ancestry specific variants associated with spontaneous preterm birth
Source: Sci Rep. 2018 Jan 9;8:226. doi: 10.1038/s41598-017-18246-5 (PMC5760643; doi:10.1038/s41598-017-18246-5)

Supplementary information

A genome-wide association study identifies only two ancestry specific variants associated with spontaneous preterm birth

Nadav Rappoport[1,2¶], Jonathan Toung[3¶], Dexter Hadley[1,2], Ronald J. Wong[3], Kazumichi Fujioka[3], Jason Reuter[4], Charles W Abbott[4], Sam Oh[5], Donglei Hu[5], Celeste Eng[5], Scott Huntsman[5], Dale L Bodian[6], John E Niederhuber[6,7], Xiumei Hong[7], Ge Zhang[8], Weronika Sikora-Wohfeld[3], Christopher R. Gignoux[4], Hui Wang[3], John Oehlert[3], Laura L. Jelliffe-Pawlowski[10], Jeffrey B. Gould[3], Gary L. Darmstadt[3], Xiaobin Wang[7], Carlos D. Bustamante[4], Michael P. Snyder[4], Elad Ziv[5], Nikolaos A. Patsopoulos[11,12,13], Louis J. Muglia[8], Esteban Burchard[5], Gary M. Shaw[3], Hugh M. O'Brodovich[3], David K. Stevenson[3], Atul J. Butte[1,2,5&*], Marina Sirota[1,2,5&*]

[1]Institute for Computational Health Sciences, University of California, San Francisco, CA
[2]Department of Pediatrics, University of California, San Francisco, San Francisco, CA
[3]Department of Pediatrics, Stanford University School of Medicine, Stanford, CA
[4]Department of Genetics, Stanford University School of Medicine, Stanford, CA
[5]Institute for Human Genetics, University of California, San Francisco, San Francisco, CA
[6]Inova Translational Medicine Institute, Inova Health System, Falls Church, VA
[7] Department of Population, Family and Reproductive Health, Center on the Early Life Origins of Disease, Johns Hopkins University Bloomberg School of Public Health, Baltimore, MD
[8]Cincinnati Children's Hospital Medical Center, Cincinnati, OH
[10]Department of Biostatistics, University of California, San Francisco, CA
[11]Systems Biology and Computer Science Program, Ann Romney Center of Neurological Diseases, Department of Neurology, Division of Genetics, Brigham & Women's Hospital, Boston MA.
[12]Harvard Medical School, Boston MA.
[13]Program in Medical and Population Genetics, Broad Institute of MIT and Harvard, Cambridge MA.
*these authors have contributed equally to this work

* Co-Corresponding Authors:
E-mail: atul.butte@ucsf.edu, marina.sirota@ucsf.edu

¶These authors contributed equally to this work.

&These authors also contributed equally to this work.

Supplementary Tables 3
Supplementary Table S1. Age groups of controls at the time of recruitment from Health and Retirement Study (HRS) 3
Supplementary Table S2. Summary of the Validation Cohorts 3
Supplementary Table S3. Regional Validation in the ITMI Cohort 5
Supplementary Table S4. Regional Validation in the FIN Cohort 5

Supplementary Figures 6
Supplementary Figure S1. Distribution of gestational age in cases 6
Supplementary Figure S2. Distribution of birth weight in cases 6
Supplementary Figure S3. Distribution of age groups in controls 7
Supplementary Figure S4. Principal components of genetic ancestry. 8
Supplementary Figure S5. Q-Q Plots in the African and Americas populations. 9
Supplementary Figure S6. Manhattan Plots in the European (EUR), East (EAS) and South Asian (SAS) populations. 10
Supplementary Figure S7. Manhattan Plots after Imputation 12
Supplementary Figure S8. PCA of each population with cases, controls and matching 1,000 Genome Project samples. 13
Supplementary Figure S9. Intensities plots for the two variants. 14

Supplementary Tables

Supplementary Table S1. Age groups of controls at the time of recruitment from Health and Retirement Study (HRS)

Age Group (years)	Male	Female	Total
<55	380	863	1243
55-59	756	1045	1801
60-64	616	1013	1629
65-69	1011	1295	2306
70-74	892	1186	2078
75-79	709	859	1568
80+	755	1127	1882
**Total**	5119	7388	12507

Supplementary Table S2. Summary of the Validation Cohorts

SNP	COHORT	**POP**	CHR	BP	A1	A2	CASE FREQ	CASE GENOTYPE COUNTS	CNTRL FREQ	CONTROL GENOTYPE COUNTS	OR	pval
**rs17591250**	**AFR DISCOVERY**	**AFR**	**1**	**238386465**	**G**	**A**	**0.1547**	**2/52/127**	**0.0633**	**3/204/1451**	**2.8140**	**4.55E-09**
**rs17591250**	**AFR DISCOVERY IMPUTED**	**AFR**	**1**	**238386465**	**C**	**T**	**0.1500**	**2/53/135**	**0.0647**	**4/207/1451**	**2.6780**	**1.65E-08**
rs17591250	AMR DISCOVERY	AMR	1	238386465	G	A	0.1760	23/213/500	0.1670	34/294/756	0.9756	0.7963
rs17591250	EAS DISCOVERY	EAS	1	238386465	G	A	0.0310	0/8/121	0.0174	0/4/111	1.3270	0.6953
rs17591250	EUR DISCOVERY	EUR	1	238386465	G	A	0.1736	4/67/145	0.1821	326/2812/6373	0.9763	0.8548
rs17591250	SAS DISCOVERY	SAS	1	238386465	G	A	0.1522	0/7/16	0.1944	3/8/25	0.7042	0.5280
rs17591250	BBC PTB	AFR	1	238386465	C	T	0.0624	3/81/613	0.0648	6/122/906	0.9607	0.9763
rs17591250	BBC SPTB	AFR	1	238386465	C	T	0.0598	2/51/407	0.0648	6/122/906	0.9177	0.6709
rs17591250	FIN Infant	EUR	1	238386465	C	T	0.2870	21/103/129	0.2750	47/216/301	1.0602	0.6188
rs17591250	FIN Mother	EUR	1	238386465	C	T	0.2710	28/125/181	0.2870	51/216/287	0.9292	0.4940
**rs17591250**	**GALA II**	**AMR**	**1**	**238386465**	**C**	**T**	**0.1254**	**7/63/237**	**0.1583**	**70/750/1992**	**0.7627**	**0.0328**
**rs17591250**	**GALA II**	**AMR**	**1**	**238386465**	**C**	**T**	**0.1254**	**7/63/237**	**0.1699**	**6/93/210**	**0.7006**	**0.0278**
rs17591250	ITMI	AFR	1	238386465	C	T	0.0484	0/3/28	0.0472	0/5/48	1.0210	0.9804
**rs17591250**	**ITMI**	**EUR**	**1**	**238386465**	**C**	**T**	**0.1220**	**1/18/63**	**0.1807**	**4/82/163**	**0.5630**	**0.0484**
rs17591250	SAGE ALL	AFR	1	238386465	C	T	0.0885	33676	0.0714	6/129/852	1.2620	0.3507
rs17591250	SAGE TAIL	AFR	1	238386465	C	T	0.0885	3/14/96	0.0874	3/33/187	1.0130	0.9637

**rs1979081**	**AMR DISCOVERY**	**AMR**	**8**	**334288**	**A**	**C**	**0.1320**	**19/147/535**	**0.2028**	**38/352/665**	**0.5656**	**3.72E-08**
**rs1979081**	**AMR DISCOVERY IMPUTED**	**AMR**	**8**	**334288**	**T**	**G**	**0.1465**	**19/177/538**	**0.1958**	**39/345/696**	**0.6755**	**6.07E-05**
**rs1979081**	**AFR DISCOVERY**	**AFR**	**8**	**334288**	**A**	**C**	**0.3344**	**24/61/78**	**0.3911**	**265/730/616**	**0.8460**	**1.76E-01**
rs1979081	EAS DISCOVERY	EAS	8	334288	A	C	0.1074	2/22/97	0.1522	1/33/81	0.7866	0.4581
rs1979081	EUR DISCOVERY	EUR	8	334288	A	C	0.1436	6/46/150	0.1575	229/2466/6587	0.8535	0.2808
rs1979081	SAS DISCOVERY	SAS	8	334288	A	C	0.2250	2/5/13	0.1667	1/10/25	1.9260	0.2985
rs1979081	BBC PTB	AFR	8	334288	T	G	0.4060	113/340/244	0.3975	154/514/366	1.0362	0.9026
rs1979081	BBC SPTB	AFR	8	334288	T	G	0.4011	74/221/165	0.3975	154/514/366	1.0151	0.7739
rs1979081	FIN Infant	EUR	8	334288	T	G	0.1230	4/54/195	0.1490	14/140/410	0.8028	0.1666
rs1979081	FIN Mother	EUR	8	334288	T	G	0.1410	4/86/244	0.1480	14/136/404	0.9404	0.6637
rs1979081	GALA II	AMR	8	334288	T	G	0.1896	5/103/190	0.1802	101/773/1832	1.0650	0.5700
rs1979081	GALA II	AMR	8	334288	T	G	0.1896	5/103/190	0.1942	8/97/186	0.9710	0.8425
rs1979081	ITMI	AFR	8	334288	T	G	0.3170	3/13/14	0.3960	8/26/19	0.8478	0.6926
rs1979081	ITMI	EUR	8	334288	T	G	0.1340	2/18/62	0.1400	3/63/181	1.0290	0.9190
rs1979081	SAGE ALL	AFR	8	334288	T	G	0.3152	8/42/42	0.3703	134/349/350	0.7826	0.1404
rs1979081	SAGE TAIL	AFR	8	334288	T	G	0.3152	8/42/42	0.3561	32/77/89	0.8325	0.3349

Supplementary Table S3. Regional Validation in the ITMI Cohort

CHR	SNP	COOR	A1	A2	P-VAL	POP	CASES	CONTROLS	OR
8	rs6996216	335118	G	C	0.04398	EUR	68/14/0	181/64/4	1.81
1	rs10737820	238386642	G	T	0.05772	AFR	2/14/15	5/25/23	0.82
1	rs111693629	238387274	A	C	0.04004	AFR	0/6/25	0/1/52	11.25
1	rs17591327	238387025	C	T	0.03348	EUR	1/17/63	4/82/163	0.60

Supplementary Table S4. Regional Validation in the FIN Cohort

SNP	CHR	COOR	DISTANCE	A1	A2	PVAL	OR/BETA	TEST
rs1979081	8	333555	733	C	A	0.0222	1.8143	Moms_logistic
rs1979081	8	333681	607	A	C	0.03193	1.7515	Moms_logistic
rs1979081	8	333797	491	T	G	0.02392	1.7993	Moms_logistic
rs1979081	8	334429	141	C	A	0.02392	1.7993	Moms_logistic
rs1979081	8	334825	537	G	A	0.02392	1.7993	Moms_logistic
rs1979081	8	335068	780	G	T	0.02392	1.7993	Moms_logistic
rs1979081	8	333313	975	G	A	0.04373	1.3966	Infants_logistic
rs1979081	8	333555	733	C	A	0.02602	-5.9242	Moms_linear
rs1979081	8	333681	607	A	C	0.03566	-5.655	Moms_linear
rs1979081	8	333797	491	T	G	0.02867	-5.825	Moms_linear
rs1979081	8	334429	141	C	A	0.02868	-5.8249	Moms_linear
rs1979081	8	334825	537	G	A	0.02868	-5.8249	Moms_linear
rs1979081	8	335068	780	G	T	0.02868	-5.8249	Moms_linear
rs1979081	8	333313	975	G	A	0.008755	-4.1976	Infants_linear
rs1979081	8	334709	421	G	A	0.01441	-3.9586	Infants_linear

Supplementary Figures

Supplementary Figure S1. Distribution of gestational age in cases

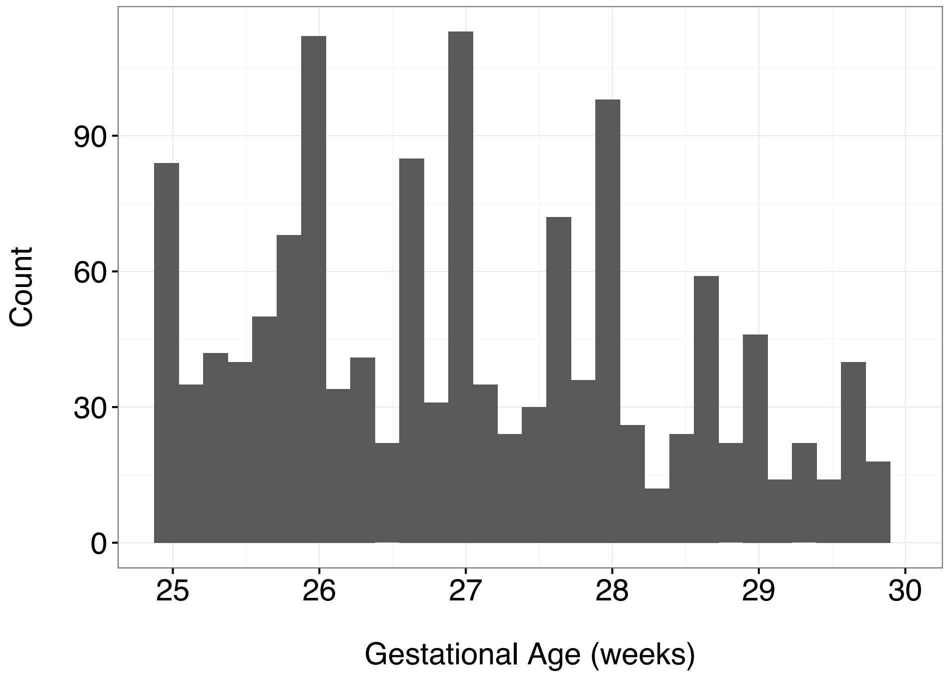

Supplementary Figure S2. Distribution of birth weight in cases

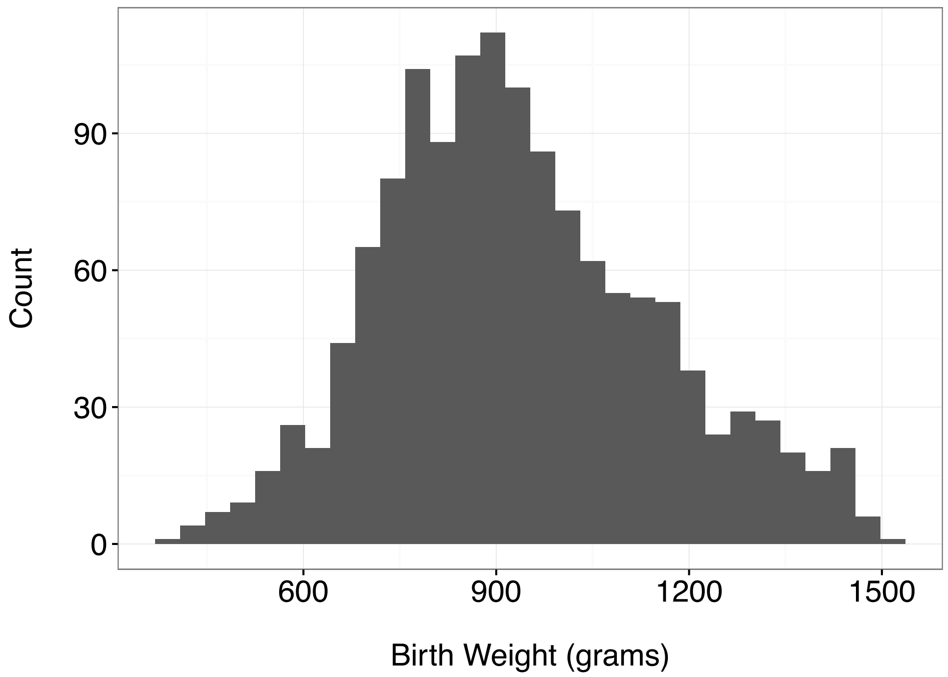

Supplementary Figure S3. Distribution of age groups in controls

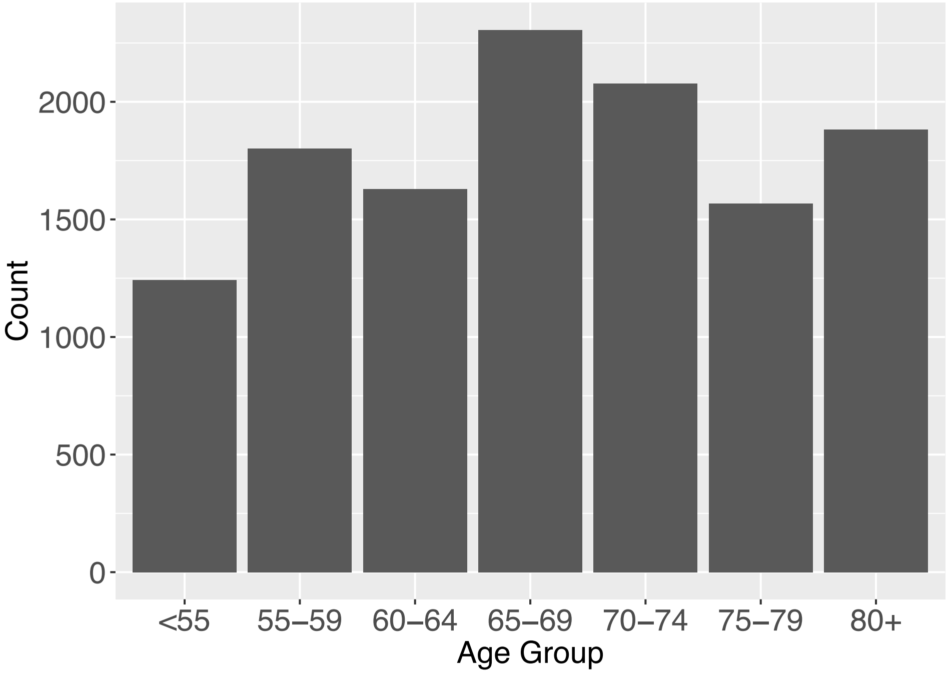

Supplementary Figure S4. Principal components of genetic ancestry.
Principal components of genetic ancestry across 15,000 individuals.} A) This is a PCA plot showing the second and third principal components of genetic ancestry colored by dataset for the three datasets in our analyses. The PTB cases are shown in red, HRS controls shown in green, and 1,000 Genomes Project shown in blue. B) This is a PCA plot similar to the one above, however colored by the five populations that we stratified our cases and controls into.

(a)

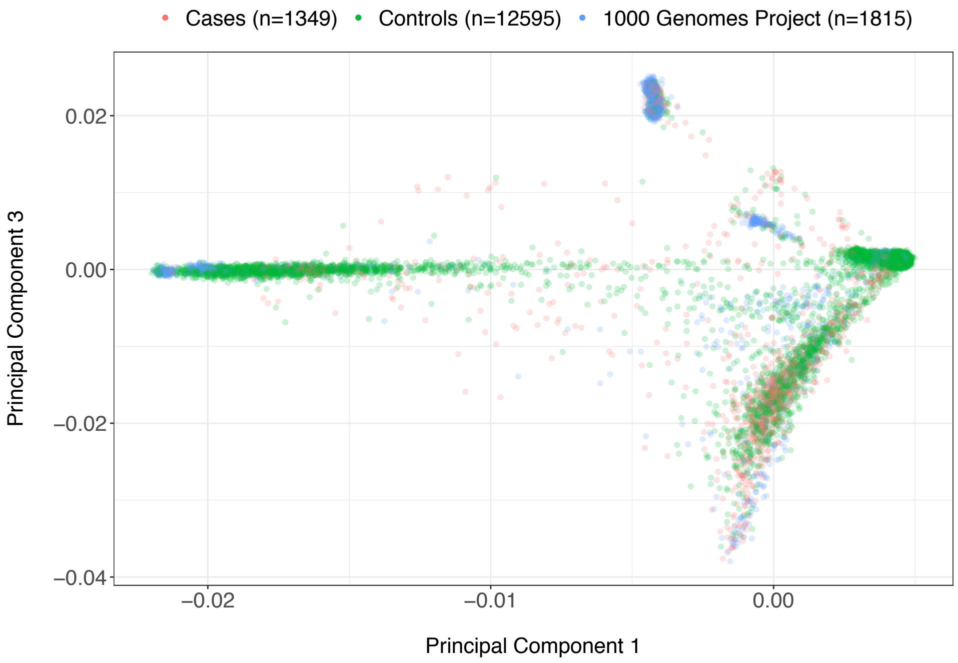

(b)

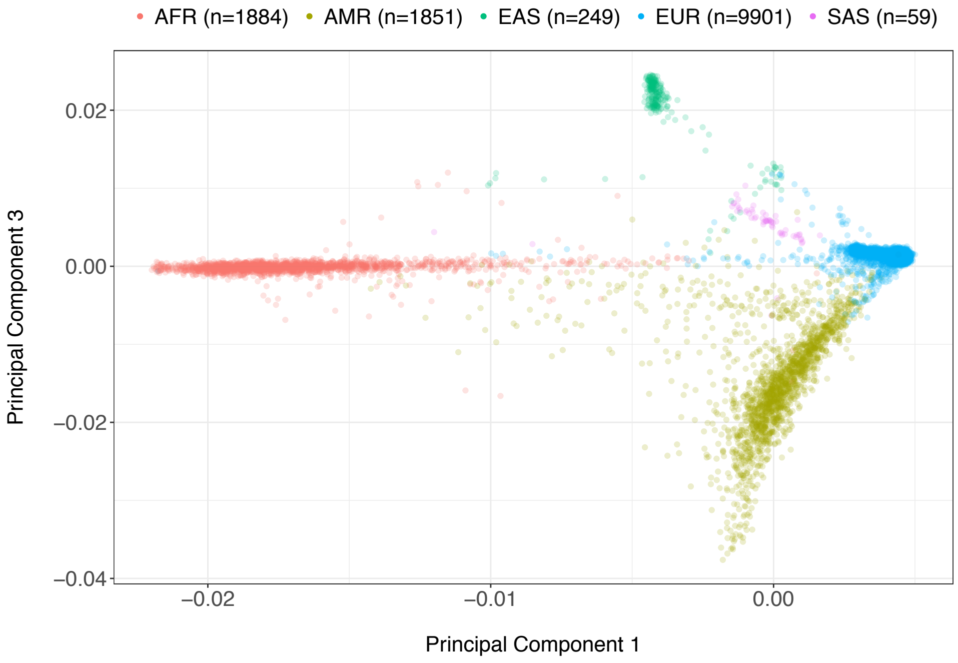

Supplementary Figure S5. Q-Q Plots in the African and Americas populations. Observed association statistics are ranked in order from smallest to largest on the y-axis and plotted against the distribution that would be expected under the null hypothesis of no association on the x-axis.

(a) AFR

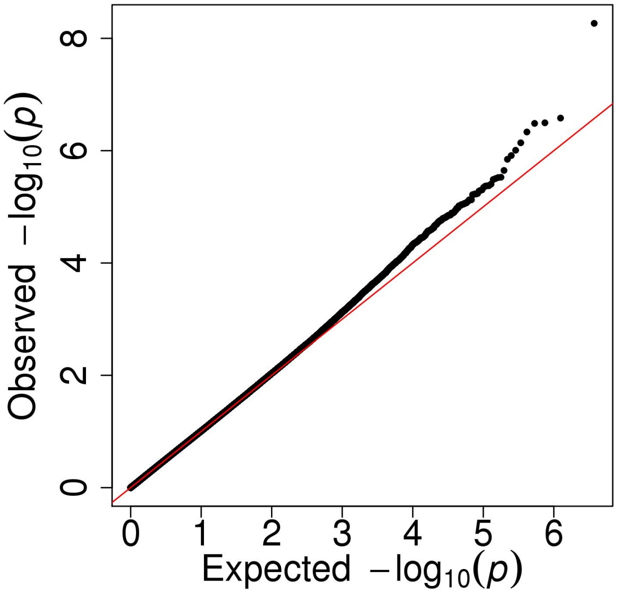

(b) AMR

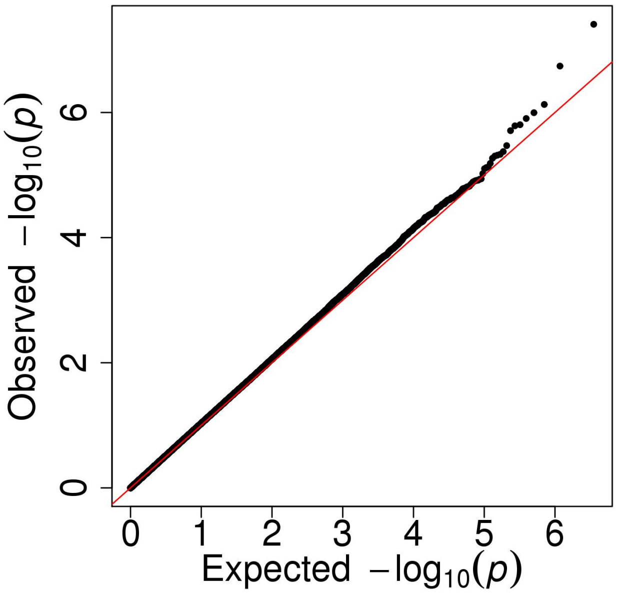

Supplementary Figure S6. Manhattan Plots in the European (EUR), East (EAS) and South Asian (SAS) populations.

These are Manhattan plots showing the results of the genotyping case control analysis in the EAS (A), EUR (B) and SAS (C) populations. The chromosomes are on the x-axis and the -log (P-value) is shown on the y-axis.

(a) EUR

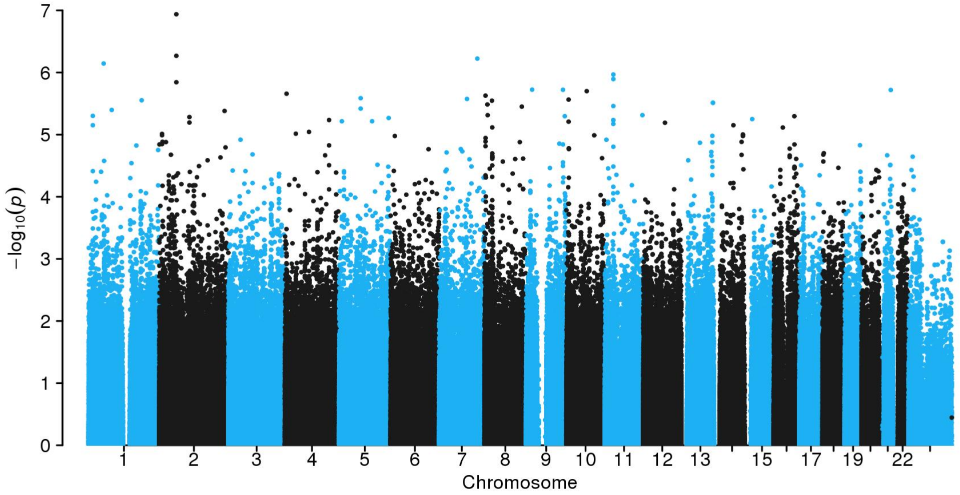

(b) EAS

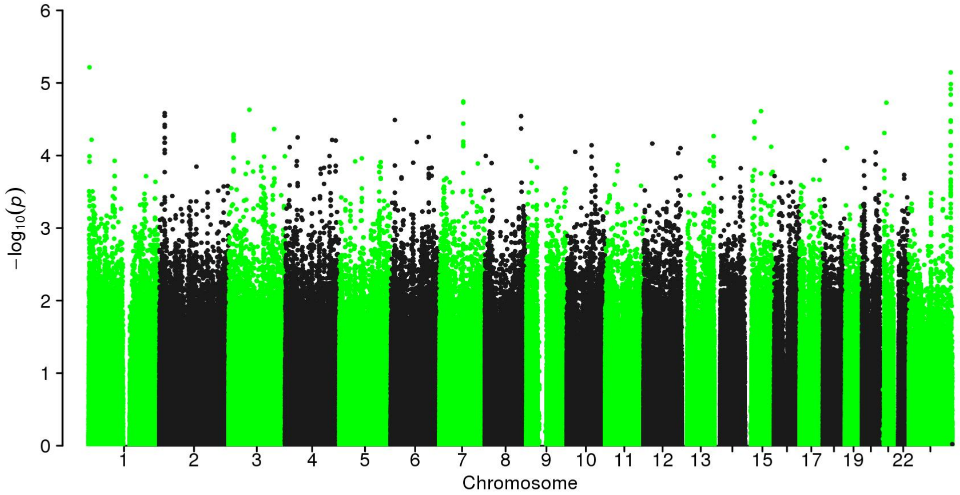

(c) SAS

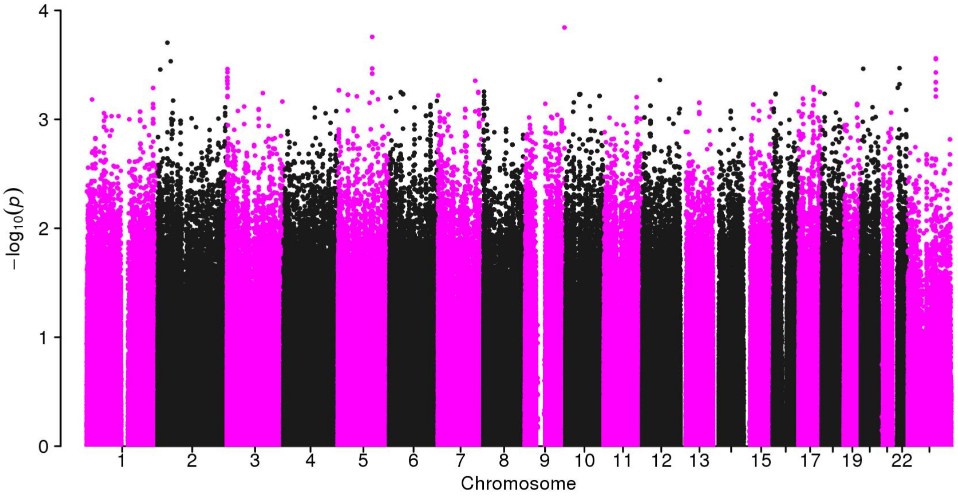

Supplementary Figure S7. Manhattan Plots after Imputation.

Regional manhattan plot ±400Kb around the variant of interest after the variant was removed and then imputed. (A) AFR population results around rs17591250 (B) AMR population results around rs1979081.

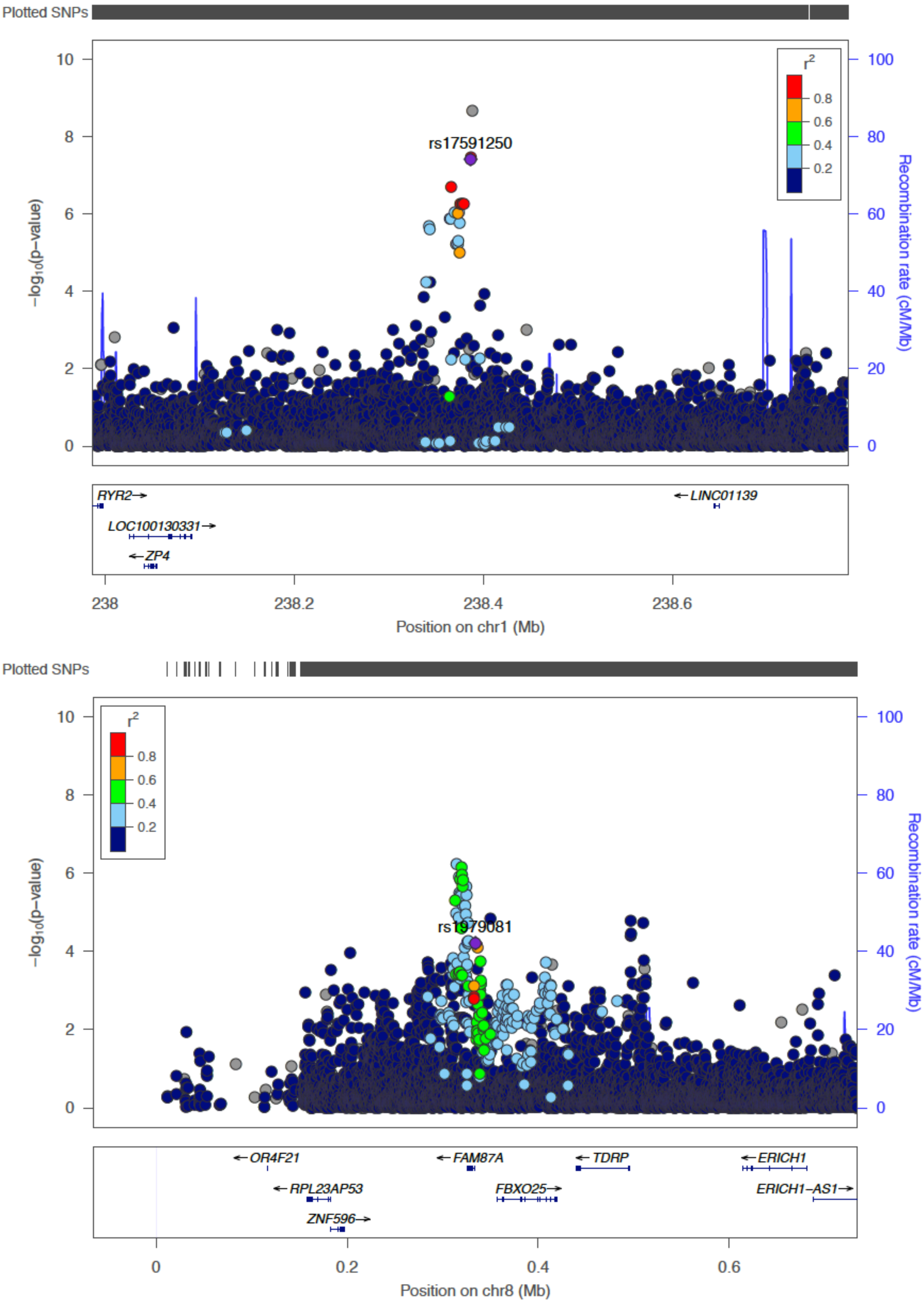

Supplementary Figure S8. PCA of each population with cases, controls and matching 1,000 Genome Project samples.

Projection of cases, controls and population matched 1KGP samples on top two principle components of (A) AFR and (B) AMR.

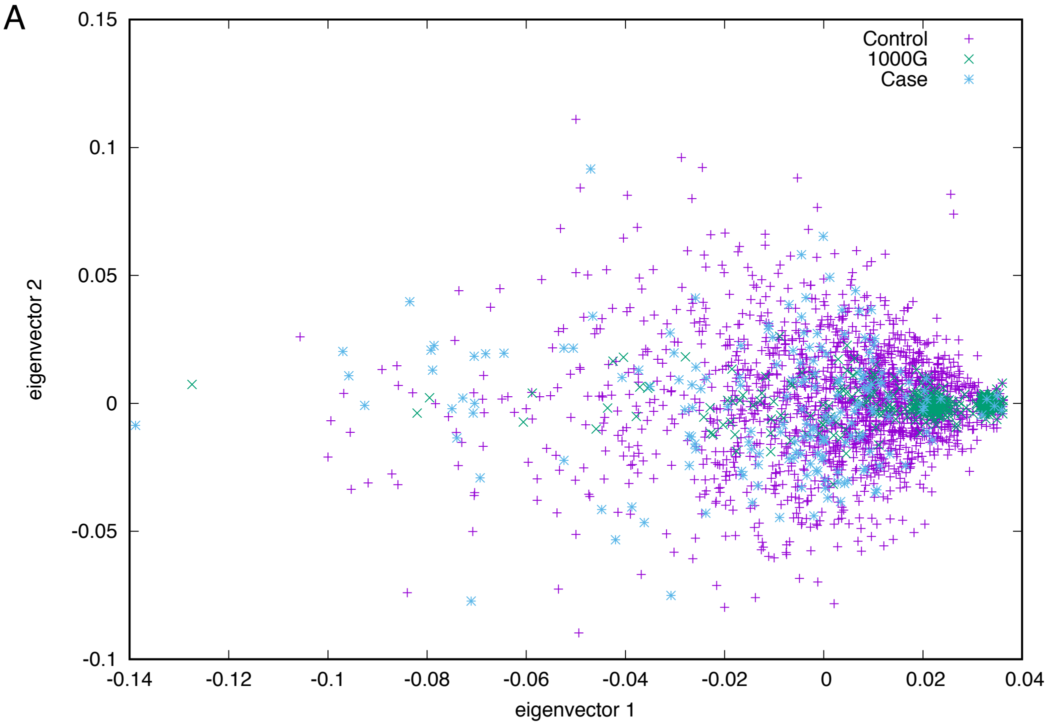

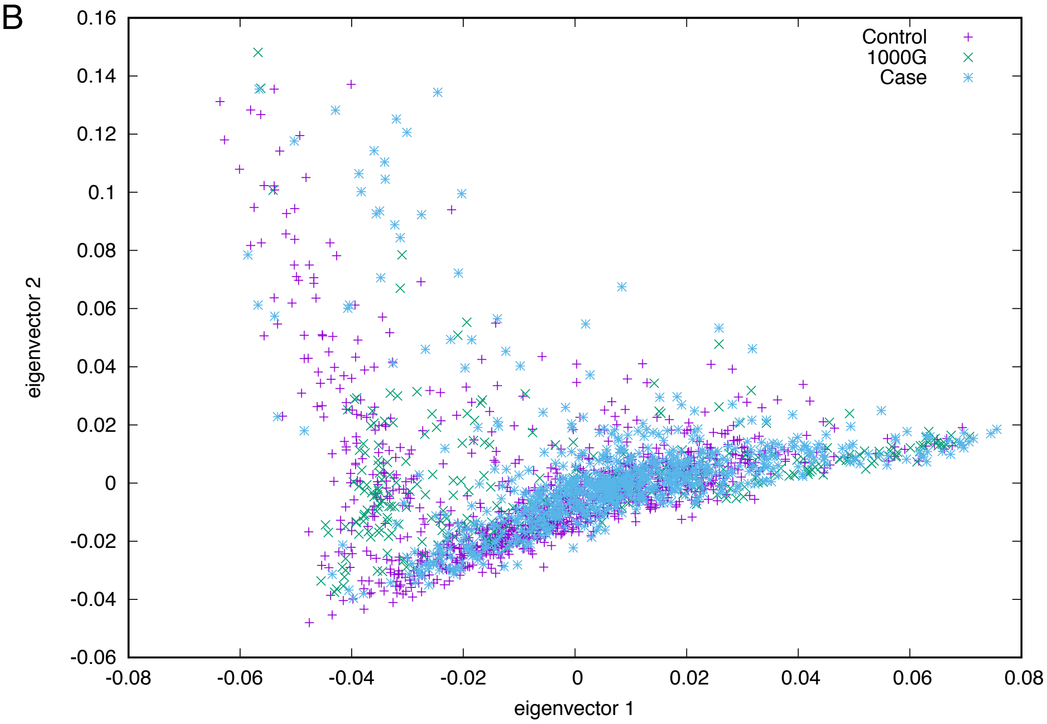

Supplementary Figure S9. Intensities plots for the two variants.
(A) AFR variant rs17591250 and (B) AMR variant rs1979081. Intensities plots are exported from Genome Studio.

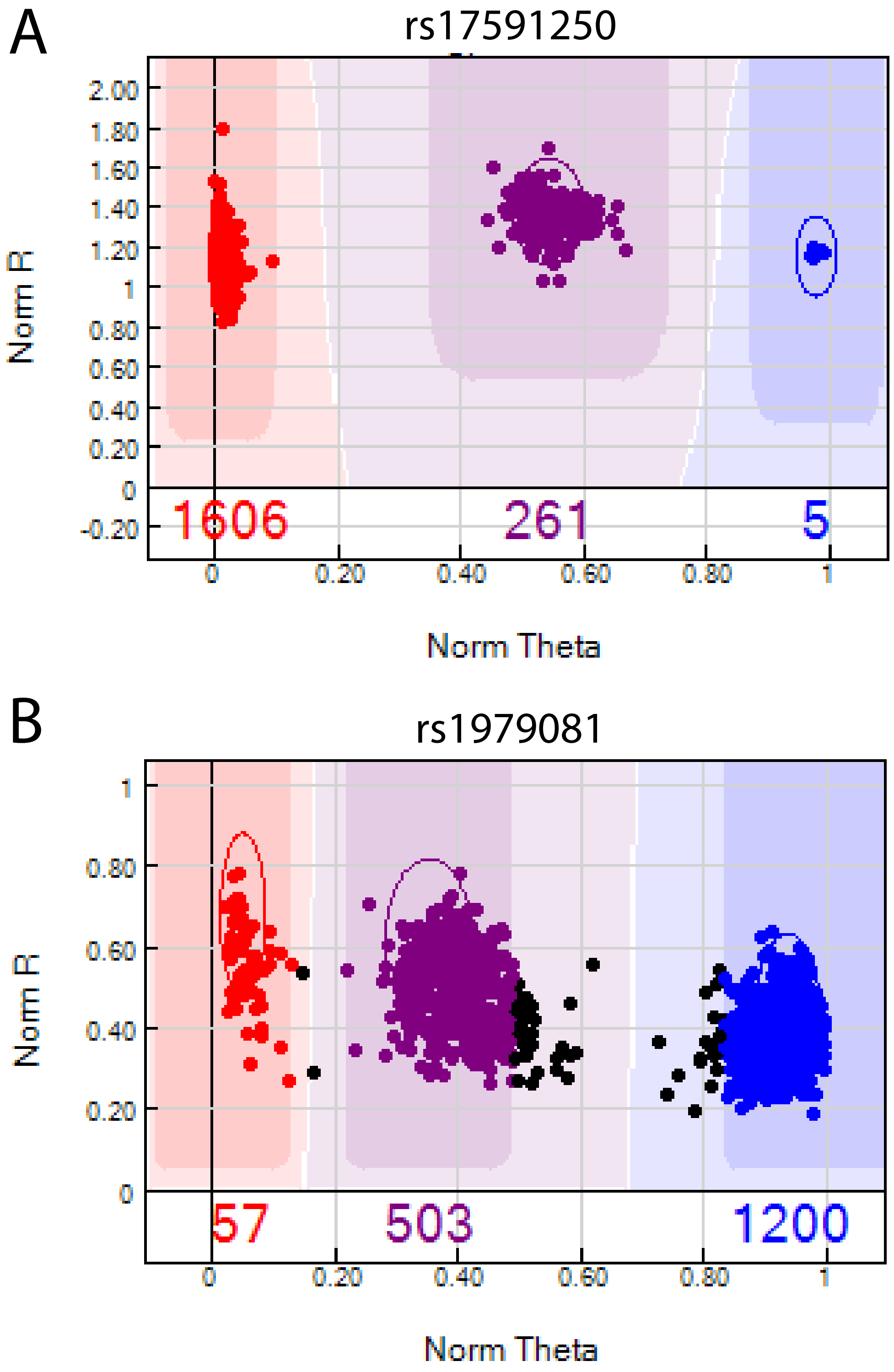

Supplement: Supplementary file 1 — Supplementary Information [file 41598_2017_18246_MOESM1_ESM.pdf]
